# The Relationship of Season of Birth with Refractive Error in Very Young Children in Eastern China

**DOI:** 10.1371/journal.pone.0100472

**Published:** 2014-06-19

**Authors:** Qinghua Ma, Wenxin Xu, Xiaohua Zhou, Chunxue Cui, Chen-Wei Pan

**Affiliations:** 1 School of Public Health, Medical College of Soochow University, Suzhou, China; 2 The 3^rd^ People's Hospital of Xiangcheng District, Suzhou, China; Zhongshan Ophthalmic Center, China

## Abstract

**Purpose:**

To determine the association of season of birth and refractive error in very young Chinese children in China.

**Methods:**

We conducted a population-based study of Chinese children aged 0 to 3 years residing in eastern China. Refraction was determined by non-cyclopegic autorefraction using a hand-held autorefractor. Date of birth was retrieved from birth certificate of the individual subjects. A generalized linear regression model was fitted to estimate the regression coefficient and 95% confidence interval (CI) of refractive error for season of birth, adjusting for confounders.

**Results:**

Of the 1385 children eligible to participate, 1222 (88.2%) were examined. Refractive error data were available for 1219 children. The mean spherical equivalent were 1.21 diopters (D) in children born Spring, 1.24 D in those born in Summer, 1.23 D in those born in Autumn, 1.15 D in Winter. After adjusting for age, sex, father's educational level, birth weight and the number of summers between birth and examination date the children have been exposed to, children born in winter had a 0.12 D more myopic refraction compared with those born in summer (regression coefficient: −0.12; 95% CI, −0.27,−0.06; P = 0.006). The association between season of birth and cylinder power was not statistically significant.

**Conclusions:**

In China, children born in winter had a more myopic refraction compared with those born in other seasons. The observed association between season of birth and refractive error was independent of parental educational level and birth weight, suggesting that light level may have a small impact on refractive development in early life.

## Introduction

Refractive error, especially myopia, is a major health concern throughout the world due to its increasingly high prevalence in the past few decades[1]. Refractive error is also associated with vision-threatening ocular complications including age-related cataract[2], age-related macular degeneration[3], open angle glaucoma[4] and diabetic retinopathy[5]. Refractive error is a complex multi-factorial trait driven by both genetic and environmental factors with environmental factors being thought to play a major role in the etiology of refractive error[6]. The major environmental risk factors related to myopia are time for near work[7]–[10] and outdoor activities[11]–[15] in childhood. Light levels have been linked to the development of refractive error but the findings were inconsistent in different studies. For example, some studies found that infants who slept with a night light or bedroom light on were at an increased risk of myopia development in later life[16]–[18]. However, other studies have failed to find an association between light exposure at night in infancy and the risk of myopia[19]–[21].

Understanding the relationship between season of birth and refractive error may provide further insights into how light levels could affect refractive status in early life. In the northern hemisphere, day lengths are relatively longer in summer and shorter in winter. Therefore, infants born during summer might be exposed to relatively higher levels of light compared with those born during winter. Up till now, only 2 studies[22]–[23] examined the association between season of birth and refractive error. McMahon et al found that birth during the summer or autumn was a significant risk factor for high myopia in subjects aged 18–100 years in the United Kingdom, increasing the risk by approximately 16% compared with subjects born in winter. However, season of birth was not a significant risk factor for low or moderate myopia[23]. Similarly, Mandel et al also reported birth during Summer was a risk factor for moderate and high myopia, but not low myopia, in young adults aged 16–22 years in Israel[22]. The major limitation for the above two studies was that the study participants were too old so that there were too many confounders which could have biased the association between season of birth and refractive error. We believe that the ideal study subjects for this research topic are children at a very young age, who have not been exposed to various environmental factors which could affect the onset and progression of refractive errors later in life. Up till now, only one study have investigated the relationship between season of birth and refractive error in young children. Deng and Gwiazda found a lower mean spherical equivalent (SE) in infants born in the summer vs. the winter and a higher percentage of myopia (SE≤−0.25 D) in infants born in the summer vs. the winter[24]. However, this study was a clinic-based design and the finding needs to be verified in a population-based sample.

In this study, we assessed the relationship between season of birth and refractive error in Chinese children aged 0–3 years in urban city in eastern China.

## Methods

### Ethnics statement

The study was approved by the Ethics Committee, Medical College of Soochow University and followed the tenets of the Declaration of Helsinki. Written informed consent was obtained from all parents after the nature of the study was explained.

### Study population

The study site was located in the Child Health Service Center in Xiangcheng District, Suzhou city, China, which provided free health screening for local children aged 0–3 years residing in this town. Before the health screening, an invitation letter was sent to each family in the town and nature of the health screening was explained in the letter. Based on the official records, there were 1385 children aged 0 to 3 years residing in the town in 2013 and 1222 (88.2%) children were screened in 2013.

### Data collection

Non-cyclopegic autorefraction was performed by a trained optometrist with a hand-held autorefractor (Retinomax K-Plus 2; Nikon, Tokyo, Japan). Sex, date of birth and birth weight were retrieved from birth certificate of the individual subjects. Date of examination was recorded and age at examination was calculated. Information on parental educational level was collected using questionnaires. Months of birth were also grouped together to divide the year into 4 seasons: winter (December–February), spring (March–May), summer (June–August), and autumn (September–November). Cover tests were performed by using fixation targets at both distance (6 m) and near (30 cm) and the presence of strabismus were recorded. Spherical equivalent (SE) was defined as sphere plus half cylinder. Myopia was defined as SE of −0.50 diopter (D) or less. Hyperopia was defined as SE of 0.50 D or more and astigmatism was defined as cylinder power less than −0.50 D.

### Data analysis

As the Spearman correlation coefficient for SE in the left and right eye was high (r = 0.88, P<0.001), only right eye data were used for analyses. SE and cylinder power were analyzed as continuous outcome variables and season of birth was investigated as categorical explanatory variables, respectively. Generalized linear regression models with data of right eyes were fitted to estimate the regression coefficients and 95% CIs of refractive error and cylinder power for season of birth, adjusting for age and gender. In multivariate analysis, we further adjusted for father's educational level, birth weight and the number of summers between birth and examination date the children have been exposed to. Statistical analyses of the data were carried out using SPSS 16.0 (SPSS Inc., Chicago, IL). P<0.05 indicated statistical significance.

## Results

**Figure 1:**
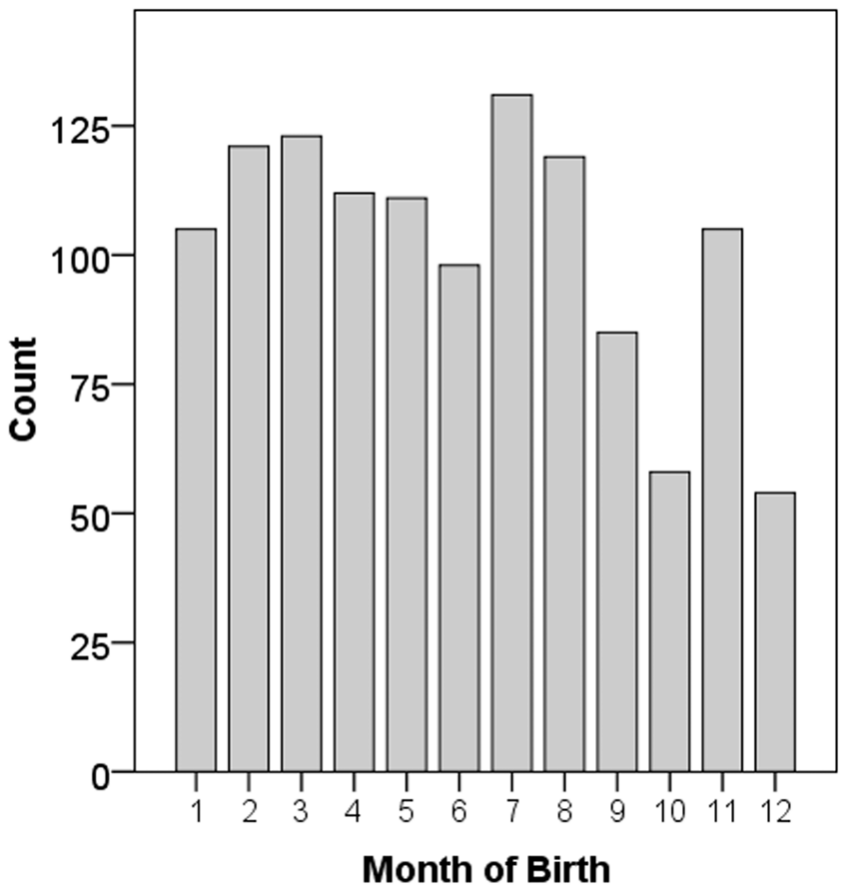
Distribution of spherical equivalents in the right eye.

**Figure 2:**
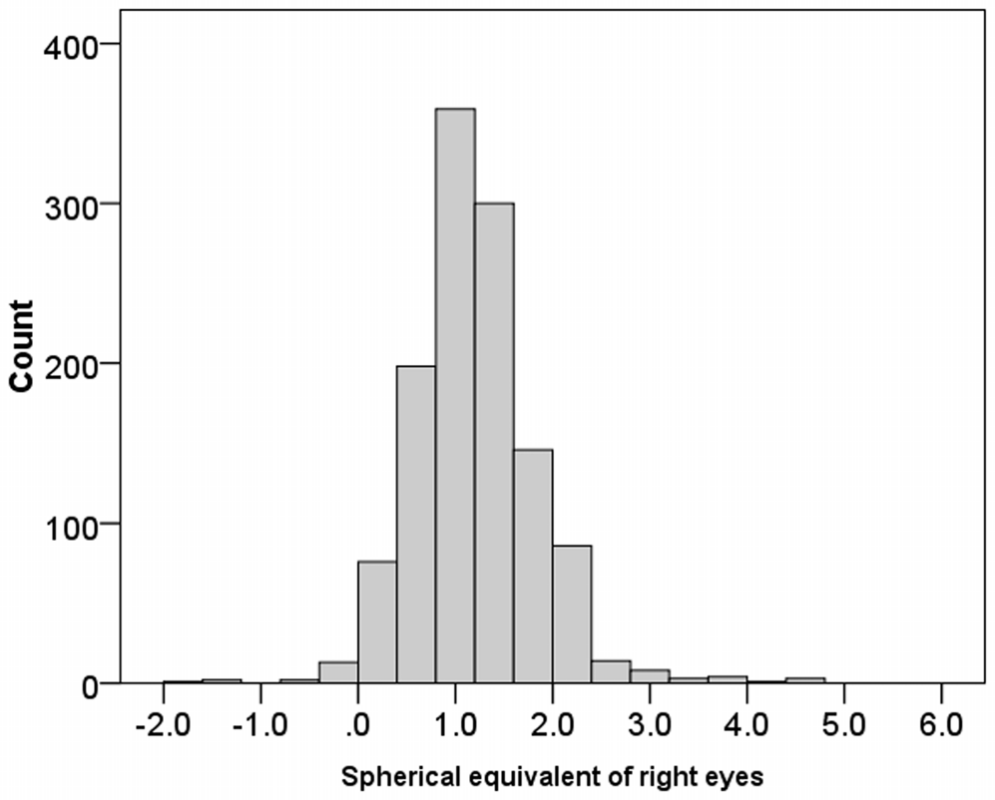
Distribution of season of birth.

Totally 661 boys and 561 girls were screened in the health service center in 2013. Three of them refused to participate in the eye screening and therefore 1219 children aged 0 to 3 years were included in the analysis. The mean age of the study subjects were 14.12±9.80 months. The mean SE were 1.26±0.73D in the left eye and 1.22±0.74D in the right eye. **Figure 1** shows the distribution of SEs in the right eye. **Figure 2** shows the distribution of month of birth in the all children. There were more children born in spring or summer than autumn or winter.

The prevalence of myopia was 0.3% in boys and 0.5% in girls. The prevalence of hyperopia was 91.2% in boys and 93.5% in girls. The prevalence of astigmatism was 74.3% in boys and 74.8% in girls. The prevalence of strabismus was 0 in the overall study sample. (**Table 1**)

**Table 1:** Prevalence of refractive errors by gender.

	Boys (n = 660)		Girls (n = 559)		P
	n	%	n	%	
Myopia	2	0.3	3	0.5	0.67
Hyperopia	603	91.2	552	93.5	0.13
Astigmatism	491	74.3	418	74.8	0.84

The mean SE were 1.21D in children born Spring, 1.24D in those born in Summer, 1.23D in those born in Autumn, 1.15D in Winter. **Table 2** shows the relationships of season of birth with refractive error and cylinder power. Children born in winter had a more myopic refraction than those born in summer after adjusting for age and sex (regression coefficient: −0.11; 95% confidence interval [CI], −0.25,−0.03; P = 0.01). After further adjusting for father's educational level and birth weight subjects born in winter had a 0.12 D more myopic refraction compared with those born in summer (regression coefficient: −0.12; 95% CI, −0.27,−0.06; P = 0.006). The association between season of birth and cylinder power was not statistically significant.

**Table 2:** Association of season of birth and refractive errors.

	Age-Gender Adjusted Models			Multivariate Adjusted Models*		
	Beta	95% CI	P	Beta	95%CI	P
Spherical equivalent						
Spring	−0.03	−0.18,0.13	0.75	−0.03	−0.19,0.13	0.59
Summer	reference			reference		
Autumn	−0.01	−0.18,0.15	0.89	−0.02	−0.19,0.17	0.82
Winter	**−0.11**	**−0.25,−0.03**	**0.01**	**−0.12**	**−0.27,−0.06**	**0.006**
Cylinder power						
Spring	−0.11	−0.26,0.04	0.16	−0.08	−0.25,0.08	0.33
Summer	reference			reference		
Autumn	0.06	−0.10,0.23	0.46	0.05	−0.14,0.24	0.45
Winter	−0.02	−0.18,0.14	0.80	−0.07	−0.23,0.10	0.49

*Models adjusted for age, sex, father's education, birth weight and the number of summers between birth and examination date the children have been exposed to.

CI = confidence interval.

## Discussion

In this study of very young Chinese children, we found that children born in winter had a more myopic refraction compared with those born in summer. The observed association between season of birth and refractive error was independent of parental educational level and birth weight. Considering that the magnitude of difference in refraction between children born in summer and winter was only about 0.1D, the results indicated that light level may have a small impact on refractive development in early life.

The association between season of birth and refractive error has been investigated in two adult cohorts and one children cohort. In Israeli-born conscripts aged 16 to 22 years, it was found that birth during summer was a risk factor for moderate and high myopia, but not low myopia[22]. In a clinic-based population aged 18–100 years, subjects born in summer or autumn were more likely to be highly myopic compared with those born in winter (summer OR = 1.17; 95% CI, 1.05–1.30; autumn OR = 1.16; 95% CI, 1.04–1.30) while season of birth was not a significant risk factor for low or moderate myopia[23]. The finding in our study was different from the previous two studies, which may be contributed to the differences in age of the study subjects between our study and the previous studies regarding the relationship between season of birth and refractive error. Our subjects were much younger than those in previous studies, which provided stronger evidences. Considering that refractive error is mainly determined by environmental factors including nearwork[9] and time outdoors[25], it is possible that other environmental factors dominating refractive error later in life have biased the results in adult studies. We believe that the age of our study subjects were ideal as they have not been exposed to any educational pressure and thus, the results were less biased. Deng and Gwiazda found that infants born in summer were a bit more myopic than those born in winter, which was not the same from our study[24]. Considering that the study was clinic-based with more potential confounders biasing the results, we think our population-based design may provide more cogent data on this research topic.

The biological mechanism linking season of birth to refractive error needs to be further discussed. Experimental and clinical results suggested that refractive development may be related to ambient light exposure[26]–[29]. The finding of our study was consistent with a study in Denmark, which showed that eye elongation and myopia progression seem to decrease in periods with longer days and to increase in periods with shorter days[30]. The day length in winter is usually 3 to 4 hours shorter than in summer in eastern China. Infants born in winter may not be exposed to ambient light compared with those born in summer. In addition, the weather in winter in eastern China is extremely cold and children spend very little time outdoors. Therefore, we think that season of birth may be a surrogate measure for time outdoors in this study.

In addition to the variation in time outdoors, season of birth reflects other population-wide changes in environmental variables including temperature, humidity, diet, sleeping time and so on. Up till now, there have been few data addressing the potential roles of these changes as risk factors for refractive error, one can only speculate which of these variables, if any, might be related to refractive error. For example, Lin et al found that higher saturated fat and cholesterol intake are associated with longer axial length in Chinese children[31]. It is likely that children may take more food with more saturated fat and cholesterol in winter due to the cold weather.

McMahon et al also suggested that the association between season of birth and refractive error may be confounded by prenatal or postnatal development such as birth weight or parental socioeconomic status[23]. Our study found that the association between season of birth and refractive error was independent of parental educational level and birth weight, providing stronger evidence on the independent association between season of birth and refractive error.

The participants of our population-based study were very young, which reduced potential biases compared with previous adult studies on this research topic. There are also some limitations of our study which should be considered. First, non-cyclopegic refraction was performed as the study subjects are too young and most parents refused the cyclopegic refraction. Non-cyclopegic refraction might have possibly overestimated the prevalence of myopia in our study but we feel that its effect on the association between refractive error and season of birth is minimal. The use of the Retinomax may cause a significant negative shift in refraction, even with cycloplegia. Secondly, we only adjusted parental educational level and birth weight in multivariate analysis. There may be other residual confounders which were not captured by our study such as parental myopia and time outdoors.

In conclusion, Chinese children born in winter had a more myopic refraction compared with those born in summer in eastern China. The observed association between season of birth and refractive error was independent of parental educational level and birth weight. These findings support that ambient light exposure may have a small impact on refractive status in early life.
